# Assessment of SNPs associated with the human glucocorticoid receptor in primary open-angle glaucoma and steroid responders

**Published:** 2010-04-03

**Authors:** John H. Fingert, Wallace L. Alward, Kai Wang, Thomas Yorio, Abbot F. Clark

**Affiliations:** 1 Department of Ophthalmology & Visual Sciences, University of Iowa, Iowa City, IA; 2 Department of Biostatistics, University of Iowa, Iowa City, IA; 3 Department of Pharmacology & Neuroscience, University of North Texas Health Science Center, Ft. Worth, TX; 4 Department of Cell Biology & Anatomy, University of North Texas Health Science Center, Ft. Worth, TX; 5 North Texas Eye Research Institute, University of North Texas Health Science Center, Ft. Worth, TX

## Abstract

**Purpose:**

While chronic glucocorticoid (GC) therapy leads to ocular hypertension in about one third of individuals, almost all primary open-angle glaucoma (POAG) patients show this response and are called “steroid responders.” Two differentially spliced isoforms of the glucocorticoid receptor (GR), GRα and GRβ, regulate GC responsiveness in trabecular meshwork (TM) cells. GRβ acts as a dominant negative regulator of GC activity and is expressed at lower levels in glaucomatous TM cells, making them more sensitive to GCs. Several arginine/serine-rich splicing factor (SR) proteins have been implicated in alternative splicing of the GR. We have previously demonstrated that immunophilins FKBP5 and FKBP4 are required for GRα and GRβ translocation into the nucleus, which is essential for their biologic activity. The purpose of the present study was to use single nucleotide polymorphism (SNP) genotyping to determine whether there are any allele frequency differences in *GR*, *FKBP4/5*, or *SR* genes between normal control, POAG, and steroid responder populations.

**Methods:**

Clinically characterized individuals (400 normal controls, 197 POAG, and 107 steroid responders) were recruited from the U. Iowa Ophthalmology Clinics after IRB approved consent. Genotyping of DNA samples for 48 SNPs in *SFRS3*, *SFRS5*, *SFRS9*, *FKBP4*, *FKBP5*, and *NR3C1* was done at GeneSeek using a mass spectroscopy based system.

**Results:**

All 48 SNPs displayed high call rates (99%). There were no significant differences in allele frequencies or genotypes in SNPs for *SFRS5*, *SFRS9*, *FKBP4*, *FKBP5*, and *NR3C1* between the 3 groups. Up to three SNPs in *SFRS3* had p-values <0.05 when comparing controls to POAG or steroid responders, but this statistical significance was lost when the p values were adjusted for multiple measures.

**Conclusions:**

Although these 6 genes may be involved in the pathogenesis of GC-induced ocular hypertension, it does not appear that major heritable risk alleles in these genes are responsible for the development of GC-induced ocular hypertension or POAG.

## Introduction

Glaucomatous optic neuropathy is a leading cause of irreversible vision loss and blindness in the world. Primary open-angle glaucoma (POAG) is one of the most prevalent forms of glaucoma, and several of the risk factors involved in POAG include family history, elevated intraocular pressure (IOP), age, race, and responsiveness to glucocorticoids (GCs). Heredity is a major risk factor in POAG [1], and several glaucoma loci have been mapped and several genes identified [2,3]. Elevated IOP, which is due to a compromised aqueous humor outflow facility at the trabecular meshwork (TM), is the primary risk factor associated with the development and progression of glaucoma [4,5].

The therapeutic use of GCs can lead to the development of ocular hypertension and iatrogenic open-angle glaucoma in susceptible individuals. This secondary glaucoma clinically mimics many features of POAG. Although only approximately 30%–40% of the normal population are “steroid responders” (i.e., develop glucocorticoid-induced ocular hypertension), most of POAG patients are steroid responders. Normal individuals who are steroid responders are at higher risk for subsequently developing POAG [6,7], and steroid responsiveness appears to also be heritable [8-10]. In addition, there have been reports suggesting that endogenous cortisol may play a role in the pathogenesis of POAG [11-13].

There are multiple isoforms of the glucocorticoid receptor (GR) [14]. GRα is the ligand binding form of the receptor that is responsible for the physiologic and pharmacological effects of GCs. GRβ is an alternatively spliced form of the receptor, which lacks the conventional ligand binding domain, does not bind GCs, and acts as a dominant negative regulator of GC activity [15,16]. Increased expression of GRβ has been implicated in the development of several steroid resistant diseases [17,18]. More germane to glaucoma, recent work in our laboratory has shown that glaucomatous TM cells have lower levels of GRβ compared to normal TM cells, and this appears to be responsible for increased GC sensitivity in the glaucomatous TM cells [19]. Levels and activities of these GR isoforms are regulated by alternative splicing as well as by nuclear translocation of the receptors. A variety of proteins and RNAs are involved in alternative splicing of gene transcripts [20], and the splicesome proteins SFRS9 [21] and SFRS5 [22] are involved in alternative splicing of the GR. In addition, the immunophilins FKBP4 and FKBP5, along with other factors such as Hsp90, are responsible for the nuclear translocation of GRα and GRβ, respectively [23].

The purpose of the present study was to determine whether common polymorphisms in several of the genes responsible for GC activity are involved in steroid responsiveness and/or POAG. Single nucleotide polymorphism (SNP) genotyping has been successfully used to identify several risk alleles and disease associated genes [24-26], so we have used this technology to evaluate potential involvement of common alleles in genes for the glucocorticoid receptor (*NR3C1*), immunophilins (*FKBP4* and *FKBP5*), and SR splicesome proteins (*SFRS3*, *SFRS5*, *SFRS9*) in the development of GC-induced ocular hypertension and POAG.

## Methods

The study was approved by the University of Iowa’s Institutional Review board and informed consent was obtained from study participants. The study included a cohort of 107 subjects that have a history of steroid-induced ocular hypertension, 197 subjects with POAG, and 400 normal control subjects that were all recruited from the Ophthalmology Clinics at the University of Iowa (Iowa City, IA). The majority of patients were Caucasian with approximately equal numbers of males and females in each group.

The cohort of glaucoma patients underwent complete ophthalmologic evaluation including slit lamp examination, Goldmann applanation tonometry, gonioscopy, perimetry, dilated stereoscopic examination of the optic nerve heads, and optic nerve head photography. Visual fields were assessed using the SITA 24–2 program on the Humphrey Field Analyzer (Humphrey-Zeiss, Dublin, CA). Patients who were unable to reliably perform automated perimetry were tested with Goldmann manual kinetic perimetry (Haag-Streit Instruments, Koeniz, Switzerland) using the Armaly-Drance protocol.

Patients were considered to have primary open angle glaucoma regardless of IOP if they had open iridocorneal angles, and evidence of glaucomatous optic nerve damage in at least one eye. Those with evidence of a secondary etiology of glaucoma such as pigment dispersion, pseudoexfoliation, inflammation, or a history of glucocorticoid therapy were excluded. Glaucomatous optic nerve damage was based on both optic nerve and visual field examination. Glaucomatous optic nerves had cup-to-disc ratios of greater than 0.7 with thinning of the neural rim, asymmetry of the optic nerve cup-to-disc ratio of >0.2, or photographic documentation of progressive loss of the neural rim. Patients were required to have visual fields of adequate quality for interpretation. For Humphrey visual fields this required a false positive rate, false negative rate and fixation loss rate of less than 33% [27]. Humphrey visual field evidence of glaucoma was based on the Collaborative Normal Tension Glaucoma Treatment Trial criteria [28]. Patients evaluated using manual kinetic perimetry were required to exhibit depression of the visual field in an arcuate pattern respecting the nasal horizontal meridian.

Corticosteroid responders included patients who exhibited an elevation of IOP of more than 5 mmHg after administration of glucocorticoid steroids (prednisolone acetate, dexamethasone, prednisolone phosphate, fluorometholone, betamethasone, or oral prednisone) for at least 4 weeks or who exhibited glaucomatous optic nerve damage (as defined above) after a prolonged course of oral or topical glucocorticoids.

The normal controls were obtained from the Comprehensive Ophthalmology Clinic at the University of Iowa. These subjects were all over age 59 and had no history of glaucoma and no family history of glaucoma. They had a normal slit lamp and optic nerve head examination. They were not tested for steroid responsiveness.

A total of 48 SNPs were selected using HapMap data to maximize the power to detect an association using the UCLA Association Study Design Server online software package. Tag SNPs were selected using HapMap data to maximize the statistical power with this software as previously described [29]. The cohorts were genotyped at 4 SNPs within *SFRS3*, 5 SNPs within *SFRS5*, 5 SNPs within *SFRS9*, 3 SNPs within *FKBP4*, 10 SNPs within *FKBP5*, and 21 SNPs within *NR3C1* using a mass spectroscopy-based system (Sequenom, San Diego, CA). Genotyping was conducted using theMassArray platform and iPlex Gold reagents with the manufacturer’s protocol by GeneSeek (Lincoln, NE). SNP allele frequencies were compared between subjects and controls using Fisher’s exact test. Genotype frequencies were compared using χ^2^ analysis. For rare variants for which the χ^2^ test was unsuitable, we used Fisher’s exact test. P-values were calculated using R 2.10.1. The Bonferroni correction was used to adjust p-values for multiple measures as needed.

Power for the current study was estimated by simulation for different values of minor allele frequency and disease odds ratio (OR) for having an additional copy of a disease allele. Simulated data were fitted using a logistic regression in which the explanatory variable is genotype coded as 0, 1, or 2. The power is computed as the proportion the slope estimate is significantly different from 0 at level 0.05 out of 10,000 simulation replicates.

## Results

A cohort of 107 subjects with a history of steroid-induced ocular hypertension (steroid-responders), 197 POAG patients, and 400 control subjects from Iowa were genotyped at a total of 48 SNPS in *SFRS3*, *SFRS5*, *SFRS9*, *FKBP4*, *FKBP5*, and *NR3C1*. Given the size of our cohort, we have adequate power under most conditions to detect powerful risk factors for the steroid response or POAG that have an odds ratio of greater than 1.75. High call rates (mean 99%) were obtained at these SNPs, which have an average spacing of 7.1 kb. Comparisons of allele frequencies and genotype frequencies of these SNPs were made between the steroid-responders and control subjects and also between POAG patients and normal subjects (Table 1). There was no significant difference in the allele frequencies or genotypes of SNPs in *SFRS5, SFRS9, FKBP5,* and *NR3C1* between patients and the control subjects (p>0.05 uncorrected for multiple measures).

**Table 1:** SNP genotyping results.

** **	** **	** **	** **	** **	**p-values (allele frequency)**		**p-values (genotype frequency)**	
**Gene**	**SNP ID**	**Location (bp)**	**Spacing (bp)**	**Minor Allele Frequency**	**NL versus Steroid Responders**	**NL versus POAG**	**NL versus Steroid Responders**	**NL versus POAG**
*SFRS3*	rs7759778	36660245	10174	0.25	0.084	0.77	**0.046**	0.10
	rs1406945	36670419	7925	0.283	**0.027**	**0.043**	**0.033**	**0.036**
	rs7344	36678344	10783	0.217	0.11	0.11	**0.011**	0.073
	rs13202984	36689127		0.292	0.15	0.27	0.33	0.44
*SFRS5*	rs7153985	69296240	9254	0.117	0.13	0.59	0.36	0.17
	rs3104	69305494	4185	0.317	0.86	0.83	0.84	0.94
	rs8019166	69309679	3579	0.195	0.34	0.86	0.26	0.74
	rs4646296	69313258	4853	0.059	0.46	0.71	0.38	0.87
	rs17556915	69318111		0.175	0.30	0.41	0.37	0.64
*SFRS9*	rs2235222	119376576	3096	0.13333	0.37	0.94	0.53	0.60
	rs3847971	119379672	4985	0.35833	0.63	0.48	0.74	0.76
	rs9040	119384657	992	0.3	0.56	0.32	0.39	0.58
	rs7027	119385649	10520	0.15	0.27	0.14	0.68	0.37
	rs540520	119396169		0.28333	0.30	0.34	0.36	0.54
*FKBP4*	rs2968909	2768125	3706	0.15833	**0.013**	0.24	0.40	0.47
	rs3759411	2771831	6156	0.1	0.051	0.51	0.87	0.67
	rs1981655	2777987		0.05833	0.22	0.73	0.18	0.72
*FKBP5*	rs755658	35657648	12970	0.05833	0.17	0.83	0.35	0.92
	rs3798346	35670618	334	0.325	0.40	0.55	0.66	0.60
	rs9366890	35670952	4108	0.175	0.92	0.87	0.96	0.97
	rs9296158	35675060	2199	0.24167	0.56	0.55	0.56	0.59
	rs4713899	35677259	6206	0.15	0.92	>0.99	0.74	0.55
	rs737054	35683465	3515	0.225	0.26	0.68	0.24	0.26
	rs3777747	35686980	10068	0.43333	0.54	0.90	0.41	0.74
	rs9380524	35697048	28515	0.14167	0.23	0.24	0.46	0.46
	rs6912833	35725563	4336	0.24167	0.88	0.46	0.18	0.67
	rs17614642	35729899		0.13333	>0.99	>0.99	0.64	0.81
*NR3C1*	rs174048	142630597	4611	0.18333	0.30	0.19	0.40	0.26
	rs17287745	142635208	2006	0.39167	0.43	0.90	0.67	0.57
	rs17287758	142637214	12202	0.15833	0.91	0.79	0.44	0.45
	rs17209251	142649416	1085	0.20833	0.52	0.77	0.75	0.88
	rs10482672	142672726	4762	0.14167	0.59	0.60	0.51	0.62
	rs33388	142677488	26078	0.45833	0.76	0.42	0.68	0.39
	rs2918418	142703566	7003	0.175	0.41	0.15	0.64	0.37
	rs4912905	142710569	25628	0.23333	0.93	0.15	0.41	0.27
	rs2963155	142736197	11736	0.3	0.11	0.34	0.19	0.13
	rs9324921	142747933	13827	0.05833	0.72	0.35	0.88	0.39
	rs10482616	142761760	10917	0.125	0.67	0.54	0.53	0.57
	rs9324924	142772677	166	0.31667	0.52	0.11	0.75	0.18
	rs7701443	142772843	2075	0.29167	0.94	0.24	0.98	0.45
	rs4244032	142774918	1807	0.21667	0.70	0.44	0.76	0.51
	rs4607376	142776725	4948	0.45	0.19	>0.99	0.30	0.73
	rs13182800	142781673	4422	0.16667	0.70	0.76	0.80	0.79
	rs12054797	142786095	1130	0.24167	0.45	0.090	0.74	0.31
	rs12656106	142787225	1915	0.3	0.62	0.15	0.85	0.25
	rs12656106	142789140	8660	0.4833	0.82	0.67	0.96	0.89
	rs12521436	142797800	699	0.175	0.36	>0.99	0.29	0.97
	rs4912913	142798499		0.4833	0.94	>0.99	0.37	0.31

When the genotype frequencies of four SNPs in *SFRS3* were compared between steroid-responders and control subjects, 3 contiguous SNPs (rs7759778, rs1406945, and rs7344) produced p-values <0.05 (uncorrected for multiple measures). Similarly, an uncorrected p-value of 0.036 was produced when the genotypes were compared between POAG patients and control subjects at one of these *SFRS3* SNPs (rs1406945). Comparison of allele frequencies at rs1406945 also produced uncorrected p-values <0.05. Finally, comparison of the allele frequencies of a single SNP in *FKBP4* (rs2968909) between steroid responders and control subjects produced a p-value of 0.013. The allele and genotype frequencies of these SNPs are shown in Table 2. However, when adjusted for multiple measures with a Bonferroni correction, none of these p-values is statistically significant.

**Table 2:** Genotype and allele frequencies of informative SNPs.

**rs7759778**								
Genotype	Norm	POAG	SR		Allele	Norm	POAG	SR
C	237	121	58		C	613	300	152
CG	139	58	36		G	177	90	60
G	19	16	12		Total	790	390	212
(blank)	5	2	1					
Grand Total	400	197	107					
**rs1406945**								
Genotype	Norm	POAG	SR		Allele	Norm	POAG	SR
C	200	88	45		C	568	261	138
GC	168	85	48		G	216	131	76
G	24	23	14		Total	784	392	214
(blank)	8	1	0					
Grand Total	400	197	107					
**rs7344**								
Genotype	Norm	POAG	SR		Allele	Norm	POAG	SR
C	12	14	11		C	156	93	53
TC	132	65	31		T	638	299	161
T	253	117	65		Total	794	392	214
(blank)	3	1	0					
Grand Total	400	197	107					

## Discussion

Glucocorticoid administration can elevate IOP in susceptible individuals that can lead to the development of an iatrogenic secondary open-angle glaucoma that mimics POAG [30]. Only a subset of normal individuals have the propensity to develop steroid-induced ocular hypertension. In contrast, most POAG patients are steroid responders, and POAG patients have also been reported to have greater sensitivity to cutaneous GC vasoconstriction [31]. However, the exact molecular mechanism(s) responsible for steroid responsiveness is currently unclear.

One potential explanation for altered GC sensitivity is inter-individual differences in the expression levels of GRβ, the dominant negative isoform of the GR. There have been numerous reports of the potential involvement of GRβ in GC resistant diseases such as inflammatory bowel syndrome, rheumatoid arthritis, and asthma, among others [17,18]. We have shown that TM cells derived from glaucomatous donors have lower levels of GRβ compared to normal TM cells and that GRβ levels regulate TM responses to dexamethasone, such as induction of myocilin, fibronectin, and GRE-luciferase as well as inhibition of TM cell phagocytosis [19,32]. Alternative splicing of GR is regulated by specific SR splicesome proteins [21,22]. In addition, translocation of both GRα and GRβ to the nucleus is essential for the biologic activities of these receptor isoforms, and immunophilins FKBP5 and FKBP4, along with other cofactors, are co-chaperones for this GRβ and GRα translocation [23].

Despite evidence implicating the involvement of GRβ in the steroid responsiveness and POAG, it does not appear that major heritable risk alleles in genes encoding GR, GR splicesome proteins, or GR nuclear translocation proteins are involved in the development of these conditions. Although there was a suggestion that genotypes and allele frequencies of *SFRS3* SNPs may be different in steroid responders and POAG patients compared to ethnically-matched controls, these allele frequencies were not statistically significant different when corrected for multiple measures. These data suggest that ancestral mutations in *SFRS3*, *SFRS5*, *SFRS9*, *FKBP4*, *FKBP5*, and *NR3C1* are not strong risk factors for disease. Our study was adequately powered to identify strong risk factors for the steroid response or POAG (odds ratio >1.75). However, the suggestive p-values obtained in our studies of *SFRS3* might be further pursued with a larger focused association study with power to detect variations that confer modest risk for steroid responsiveness or POAG. Also, it remains possible that non-ancestral variations in these genes (that cannot be detected by association studies) may confer risk for steroid responsiveness or POAG.

Another recent study examined GR polymorphisms in patients who had received intravitreal triamcinolone injections [33]. There were no statistically significant associations between any of the 6 tested GR polymorphisms and the magnitude of IOP elevation in these patients. However, only 52 patients were evaluated in this study, some of whom were steroid responsive and others non-responsive (although the number of responders was not disclosed), so only a very strong risk correlation would have been identified. In our study, we examined a larger number of characterized steroid responders with 21 SNPs spanning the *GR* gene, and we also did not find an association. However, these two studies differ in several ways. In our study, we had more steroid responders (n=107), but we did not have access to significant numbers of clinically characterized nonresponders. We therefore had to compare the steroid responder population with the normal control population, a significant minority of which are most likely untested steroid responders (approximately 30%), which makes it more difficult to see a correlation between these two groups.

Currently, the propensity to develop GC-induced ocular hypertension must be determined empirically. Therefore, all patients on protracted GC therapy should have their IOPs monitored periodically throughout the course of GC therapy. In addition, patients who are documented steroid responders have a higher risk for developing POAG. Therefore, there is a definite need for a reliable test that would predict steroid responsiveness in patients. Unfortunately, it does not appear that genetically screening for the SNPs in the genes evaluated in our study will be useful markers to predict steroid-induced ocular hypertension or POAG, at least in the current population studied.
